# Post traumatic left cardiac luxation: A case report

**DOI:** 10.1016/j.ijscr.2020.04.095

**Published:** 2020-05-15

**Authors:** Mokhles Lajmi, Imen Ben Ismail, Wafa Ragmoun, Houssem Messaoudi, Hatem Lahdhili, Slim Chenik

**Affiliations:** a Department of Cardiac and Thoracic Surgery, The Military Hospital of Tunis, Tunisia; b Department of General Surgery, Traumatology and Severe Burns Center Ben Arous, Tunisia

**Keywords:** Pericardial rupture, Cardiac luxation, Median sternotomy

## Abstract

**Introduction:**

Rupture of the pericardium complicating blunt thoracic trauma may lead to cardiac luxation if not early recognized and appropriately surgically treated. This condition is exceedingly rare and carries a high mortality rate.

**Case presentation:**

A 37-year-old man presented with severe multiple injuries following a road traffic crash, including blunt trauma to the thorax and abdomen. He was in a hemodynamically stable condition. The chest radiograph and CT scan revealed a leftward heart herniation through a pericardial tear, there were also non surgical hepatic and splenic lesions. The diagnosis of left cardiac luxation with no associated valvular lesions was made. Surgical exploration via a midline sternotomy revealed a protruding heart herniating into the left pleural cavity through a large tear (15 cm) of the left pericardium. Sutures with Teflon felt pledgets were performed. The postoperative chest radiograph was normal and the postoperative course was uneventful.

**Discussion:**

Post traumatic heart luxation diagnosis is clinically difficult because the scarcity of signs and lack of specificity. Chest X ray can be suggestive, and specific findings can only be detected by computed tomography, which is the most sensitive diagnostic method. Surgical management in this condition is mandatory to avoid fatal complications such as great vessels strangulation. It consists of replacing the heart in the pericardial sac and repairing the pericardial tear.

**Conclusion:**

Despite its rare occurrence, trauma surgeons must be aware of cardiac luxation. Any suspicion of such a diagnosis would warrant surgical exploration to avoid fatal complications.

## Highlights

• Luxation of the heart after blunt thoracic trauma is an exceedingly rare condition.
• Traumatic pericardial rupture results in luxation of the heart.
• If undetected it has a high mortality rate.
• CT scan is the diagnostic method of choice in hemodynamically stable patients.
• Surgical approach is dictated by the surgeon's experience and associated lesions.

## 1 Introduction

Rupture of the pericardium with luxation of the heart after blunt thoracic trauma is an exceedingly rare condition with a reported incidence of 0.37 % [1]. But the true incidence may be underestimated since cardiac luxation carries a high mortality rate ranging from 30 to 67 % [2] because of quickly evolving hemodynamic failure and associated high-energy trauma, often causing multiple concomitant injuries [2]. The diagnosis of this condition is difficult and often incidentally made at emergency thoracotomy. Furthermore, the routine use of computed tomography (CT) scan in the management of patients with trauma might establish the early diagnosis of cardiac luxation. Surgery can be deemed the gold standard in the treatment of this lifethreatening injury. The time interval for the diagnosis of cardiac luxation is the most significant prognostic factor, and survival is often limited by associated injuries [2].

We herein report a case of left luxation of the heart in a young patient with multiple injuries due to an automobile accident.

This work has been reported in line with the SCARE criteria [3].

## 2 Case presentation

We report the case of a 37-year-old man presented with severe multiple injuries following a road traffic crash, including blunt trauma to the thorax and abdomen. He was in a hemodynamically stable condition.

**Fig. 1:**
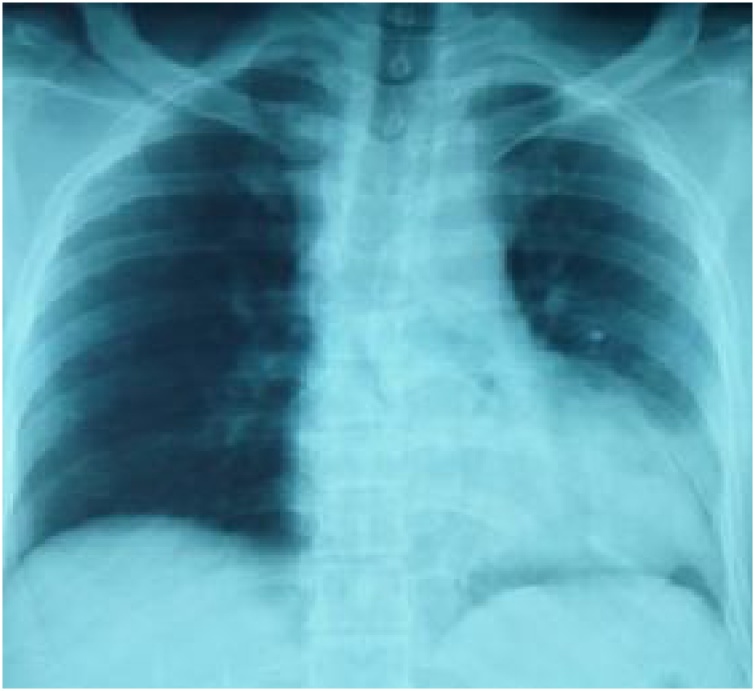
Chest radiography showing leftward deviation of the cardiac silhouette.

**Fig. 2:**
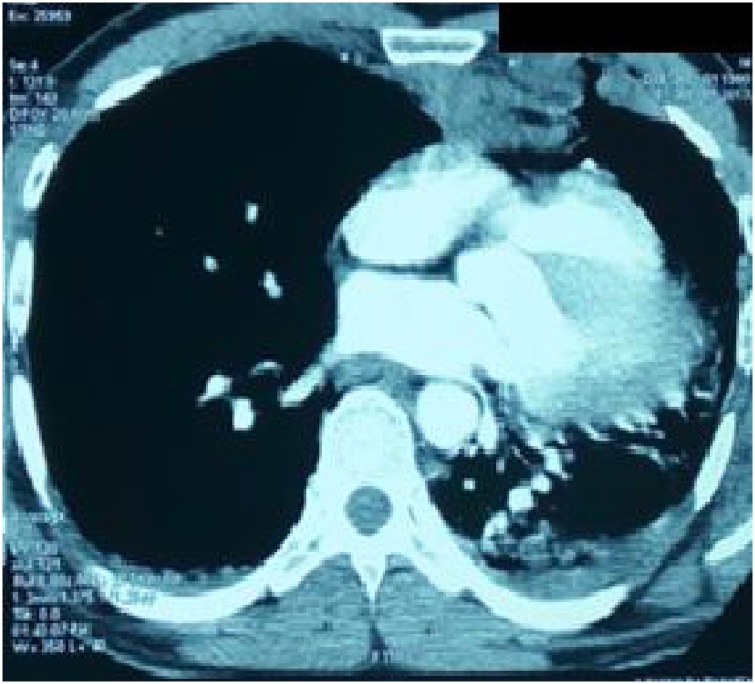
Computed tomogram shows cardiac luxation with displacement of the heart into the left hemithorax and atelectasis of the lower lobe of the left lung.

The chest radiograph revealed a leftward deviation of the cardiac silhouette (Fig. 1). The findings of the electrocardiogram were normal, except for a sinus tachycardia. Given the hemodynamic stability and the availability of an early CT scan, FAST was not performed. A total body CT scan revealed a moderate left anterior pneumothorax, a negligible right pneumothorax, fracture of the middle arch of the 5th, 6th, seventh and eighth left ribs and a fracture of the sternal manubrium. However, the heart silhouette shifted on the left pleural space, demonstrating rupture of the pericardium complicated with left cardiac luxation (Fig. 2). There were no cardiac cavity or great vessel strangulation signs. Moreover in the abdomen, there were contusions of segments 4 and 7 of the liver of 10 and 18 mm respectively, as well as a lower polar splenic contusion and an intraperitoneal effusion of medium abundance. These hepatic and splenic contusions were managed conservatively.

The diagnosis of left cardiac luxation was made, and surgery was then performed on an emergency basis.

Echocardiography was performed in the operating room just before the surgery and was normal, notably there was no valvular injuries.

**Fig. 3:**
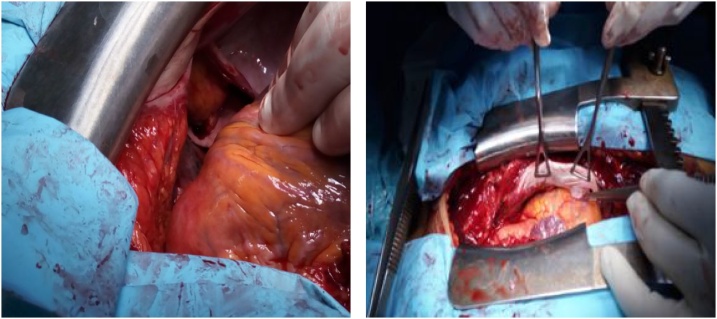
Intraoperative photographs showing the pericardial tear.

Surgical exploration via a midline sternotomy revealed the heart herniated into the left pleural cavity through a large tear (15 cm) of the left pericardium bound by the left phrenic nerve and the left pulmonary veins (Fig. 3).

Undertaking the repair was challenging. A short interval of time was available between each exposure of the tear, causing hemodynamic impairment due to heart manipulation. Sutures with Teflon felt pledgets were performed.

Close collaboration with the anesthesiologist allowed maintaining adequate blood pressure throughout the procedure, and no cardiac arrhythmia occurred.

We managed to avoid cardiopulmonary because systemic heparinization would have involved a high risk of massive hemorrhage from the patient’s intra-abdominal lesions.

**Fig. 4:**
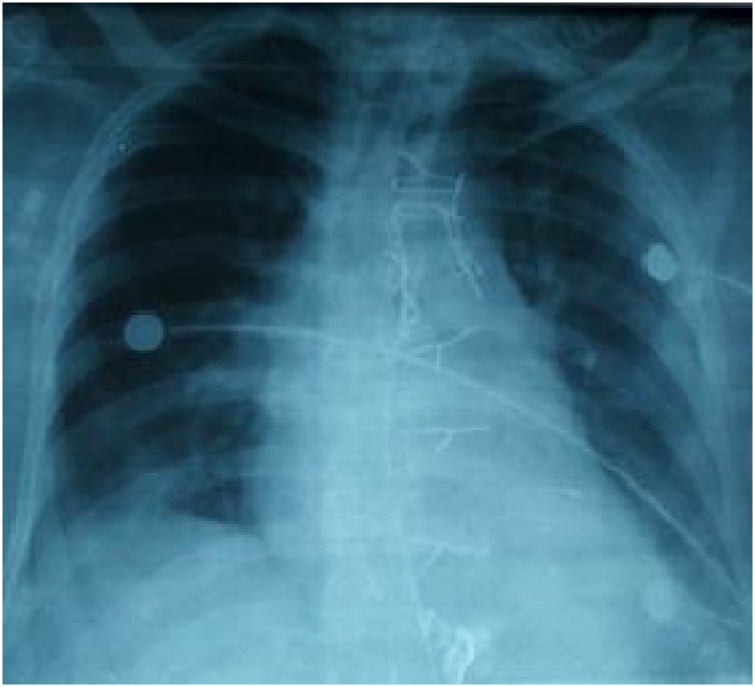
Normal postoperative chest radiograph.

The postoperative chest radiograph was normal (Fig. 4). The postoperative course was uneventful, and the patient was discharged on the 7th postoperative day. At his 1-year follow-up visit, thes patient was asymptomatic and chest x-ay and echocardiography did not reveal any abnormality

## 3 Discussion

The rupture of the pericardium is a rare complication of blunt chest trauma (occurring in approximately 1 %–2 % of cases) [4]. Its diagnosis is challenging. If the latter is delayed, it may lead to life-threatening complications like cardiac luxation.

Left-sided herniation occurs more frequently than right-sided ones. Thus, more than 60 % of all pericardial ruptures are located in the left hemithorax [1].

Regarding the mechanism, ruptures of the heart and pericardium from the blunt trauma of the chest are due, in more than 60 % of cases, to automobile accidents [5].

Post-traumatic cardiac luxation is subdivided into primary heart dislocations (occurring directly after trauma) and secondary heart dislocations (occurring weeks after trauma) [6], accounting for 65.3 % and 34.7 % respectively [7].

The diagnosis of this entity is rarely assessed clinically. The most described clinical signs resemble those of tamponade, including fluctuating hemodynamic values and a splashing millwheel murmur [2]. In our case, the patient was in a hemodynamically stable condition.

The electrocardiogram can sometimes be helpful by showing nonspecific modifications such as axial deviation and ST-segment changes [8].

Early diagnosis of post-traumatic heart luxation may be guided by chest X-ray. Nevertheless, the latter often shows only trivial features of dislocation, such as an abnormal cardiac silhouette [9] and mediastinal displacement, which can be overlooked [10].

However, specific findings, such as pneumopericardium and pericardial dimpling or discontinuity, can only be detected by computed tomography, which is the most sensitive diagnostic method [6].

As traumatic pericardial tears can be associated with injuries of the myocardium and valves. Clark et al. [1] reported tricuspid valve injuries in 3% of patients. Echocardiography is necessary to visualize associated heart damage. However, it seldom contributes to diagnose a pericardial rupture accurately [11].

It is essential to keep in mind that cardiac luxation can be intermittent; that is why sequential evaluation over time is recommended [12].

Surgical management in this condition is mandatory to avoid fatal complications. It consists of replacing the heart in the pericardial sac and repairing the pericardial tear.

Methods of pericardial repair include direct suture repair, pleural patch repair, and Teflon mesh [13]. In a systematic review of the literature Graef et al. demonstrated that closure of the pericardial tear was done via sutures in 27 %, via patch-plastic in 35 % and in 12 % of cases, the tear was left open [7].

Although scientific evidence on this subject is controversial, it seems that small defects can be primarily closed with nonabsorbable sutures; however, large defects need to be closed with prosthetic patches [2].

In the emergency setting, it has been shown that left- or right-sided thoracotomy or sternotomy are adequate approaches. The choice of the surgical approach is left to the surgeon depending on his experience [7]. Furthermore, unilateral thoracotomies have been favorably used. Graef et al. showed that pericardial tears or cardiac dislocations in the left hemithorax were treated operatively via left-sided thoracotomy in 83 % of cases and via sternotomy in 13.8 % of cases [7]

We opted for sternotomy to avoid proceeding through a contuse hemithorax with multiple rib fractures and to stabilize at the same time the sternum fracture, although it would not justify a surgical treatment for this sole reason. The obesity of the patient justified our choice as we saw median sternotomy more apt and easy to perform

Nonetheless, surgical exposure was harder than anticipated, made difficult because of the quick hemodynamic impairment after each heart manipulation. The fact that the tear was rather posterior than lateral and its width could explain those difficulties. In this setting, left thoracotomy seems a better option, offering good exposure without any impact on hemodynamics.

As these trauma complications are rare, the surgical experience is of paramount importance with choice of surgical approach individualized based on the location of injury and experience of the surgeon.

Nonoperative treatment of traumatic pericardial ruptures and cardiac luxation is exceptional. Only two cases have been reported in the literature. Poletti et al. [14] reported the case of 22-year-old male patient who presented with an asymptomatic traumatic pericardial rupture with partial right atrial herniation secondary to a high-speed motor vehicle crash. This injury was successfully managed nonoperatively. The second case was reported by Nguyen et al. in 2018 [15]. A 38-year-old men presented with post traumatic right-sided cardiac luxation with right pneumothorax. The heart moved back into the pericardium after lung re-expansion due to evacuation of the pneumothorax.

Recently Graef et al. [7] proposed a classification system for cardiac luxation, based on radiologic and intraoperative findings, in order to provide a starting point for future standardization of the diagnosis and recommended treatment. The classification includes 3 grades of increasing severity : I, pericardial rupture without dislocation of the heart; II, pericardial rupture with subluxation of the heart; III a, complete dislocation of the heart outside of the pericardium; III b, complete dislocation of the heart and malrotation; III c, complete dislocation of the heart in combination with a myocardial tear/rupture.

We have to insist on the fact that these are mostly polytrauma patients and that the cardiac luxation, especially in stable patients, must not overshadow other potentially serious lesions. Efficient reanimation is the key, and time is of the essence, as the result of surgical intervention is related to the severity and prompt treatment of other associated injuries [16].

## 4 Conclusion

Despite its rare occurrence, thoracic surgeons must be aware of cardiac luxation. CT plays a crucial role in the early diagnosis. Any suspicion of such a diagnosis would warrant surgical exploration to avoid fatal complications.

## Declaration of Competing Interest

Authors declare no conflict of interest.

## Funding

This study was not supported by any institution and company.

## Ethical approval

Ethical approval was not required and patient identifying knowledge was not presented in the report.

## Consent

Written informed consent was obtained from the patient for publication of this case report and accompanying images. A copy of the written consent is available for review by the Editor-in-Chief of this journal on request.

## Author contribution

Manuscript writing: Dr Ben Ismail Imen.

Study concepts: Dr Lajmi Mokhles, Dr Messaoudi Houssem.

Helped in data interpretation and manuscript evaluation: Dr Lahdhili Hatem.

Data acquisition: Dr Ragmoun Wafa.

Critical revision: Dr Chenik Slim.

## Registration of research studies

1 Name of the registry:
2 Unique identifying number or registration ID:
3 Hyperlink to your specific registration (must be publicly accessible and will be checked):

## Guarantor

Dr Chenik Slim.

## Provenance and peer review

Not commissioned, externally peer-reviewed.
